# Size adaptation: Do you know it when you see it?

**DOI:** 10.3758/s13414-024-02925-3

**Published:** 2024-07-29

**Authors:** Sami R. Yousif, Sam Clarke

**Affiliations:** 1 Department of Psychology, University of Pennsylvania, 425 S. University Ave, Stephen A. Levin Bldg., Philadelphia, PA 19104-6241 USA; 2 Department of Philosophy, University of Southern California, Los Angeles, CA USA

**Keywords:** Visual perception, Spatial cognition, Adaptation and Aftereffects

## Abstract

The visual system adapts to a wide range of visual features, from lower-level features like color and motion to higher-level features like causality and, perhaps, number. According to some, adaptation is a strictly perceptual phenomenon, such that the presence of adaptation licenses the claim that a feature is truly perceptual in nature. Given the theoretical importance of claims about adaptation, then, it is important to understand exactly when the visual system does and does not exhibit adaptation. Here, we take as a case study one specific kind of adaptation: visual adaptation to *size*. Supported by evidence from four experiments, we argue that, despite robust effects of size adaptation in the lab, (1) size adaptation effects are phenomenologically underwhelming (in some cases, hardly appreciable at all), (2) some effects of size adaptation appear contradictory, and difficult to explain given current theories of size adaptation, and (3) prior studies on size adaptation may have failed to isolate size as the adapted dimension. Ultimately, we argue that while there is evidence to license the claim that size adaptation is genuine, size adaptation is a puzzling and poorly understood phenomenon.

## Introduction

**Fig. 1:**
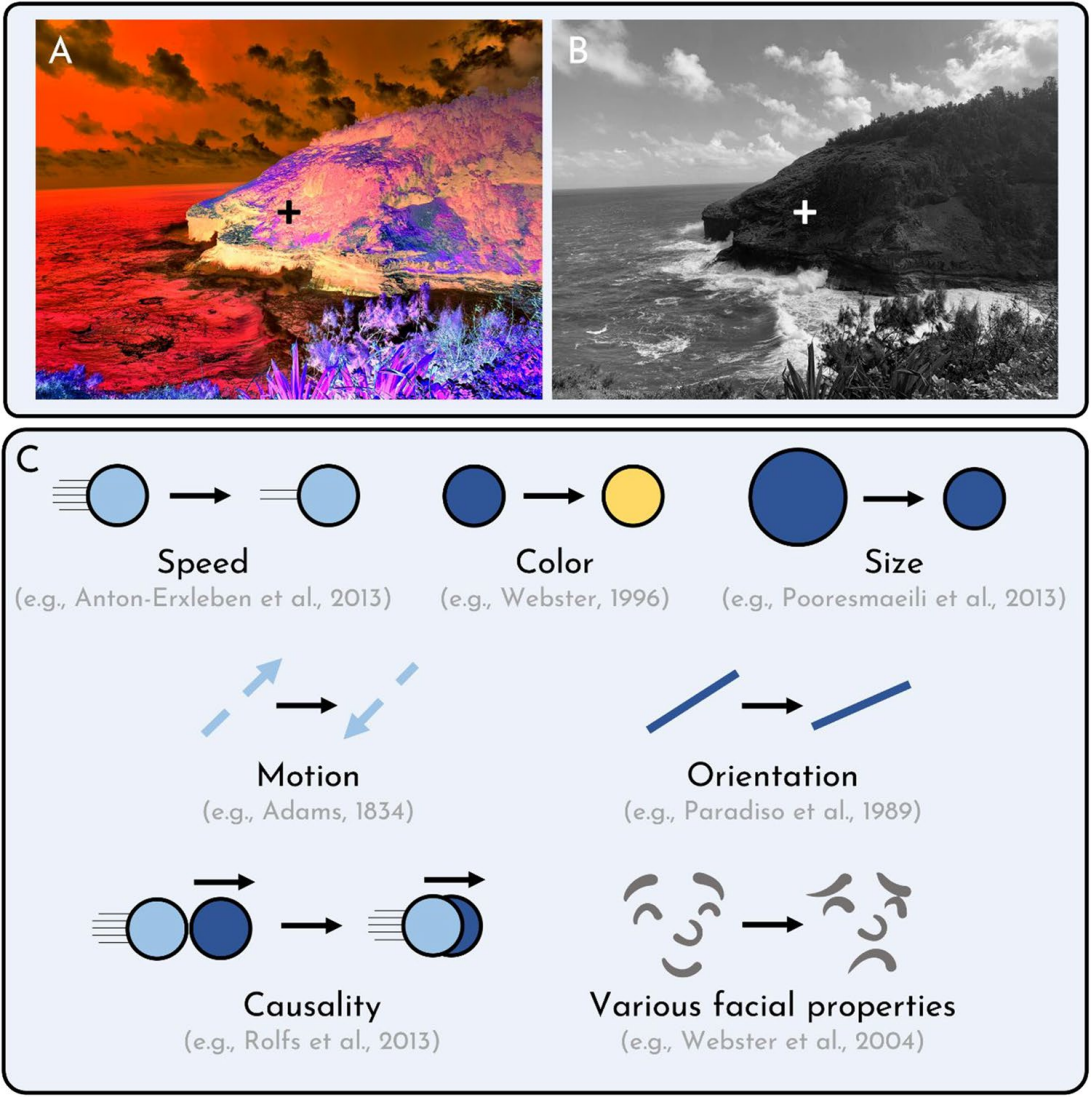
Examples of adaptation effects. If you stare at the fixation cross in (**A**) for about 20 s and then shift your focus to (**B**), you will see a colorful image, despite the fact that, as you can see, there is no color in (**B**). This is an example of color adaptation. There are many other varieties of visual adaptation effects, which are depicted in (**C**)

Take a moment to investigate Fig. 1. In the first panel (A), you will see an oddly colored image with a central fixation cross. Stare at that cross. After about 20 s, move your eyes to the fixation cross in the greyscale image beside it (B). You will see something remarkable: A vivid, colorful display, where no colors are present. (This demo is even more powerful when you keep your eyes in place; you can see a stronger version in Demo #1 of our Online Supplementary Materials (OSM).)

What you have just experienced is *color adaptation*, a canonical kind of adaptation that belongs to a broader class of adaptation effects. To date, there have been documented effects of adaptation to countless visual features (see Fig. 1C), including color (McCollough, 1965; Webster, 1996), contrast (Webster & Miyahara, 1997), orientation (Knapen et al., 2010; Paradiso et al., 1989), motion (Bartlett et al., 2019; Winaer et al., 2008, 2010), speed (Anton-Erxleben et al., 2013), and even higher-level visual features like facial properties (e.g., emotion, gender, race, etc.; Webster & MacLeod, 2011), gait (Jordan et al., 2006), number (Burr & Ross, 2008), causation (Kominsky & Scholl, 2020; Rolfs et al., 2013), and, the focus of this paper, *size* (Kreutzer et al., 2015; Pooresmaeili et al., 2013; Tonelli et al., 2020; Zeng et al., 2017). In fact, adaptation is not just a visual phenomenon; there are many documented cases of adaptation in other sensory modalities, including touch (Calzolari et al., 2017), olfaction (Dalton, 2000), and taste (McBurney & Pfaffmann, 1963; McBurney et al., 1972).

Such effects are more than a mere curiosity. In the philosophy and psychology of perception, adaptation effects are often taken to *mark the perceptual*; they are viewed as a near-definitive test of the boundaries between seeing and thinking (Block, 2022). That is: Any feature representation that exhibits adaptation is said to be perceptual in nature on the grounds that (a) adaptation is pervasive in perceptual processing and (b) rare or absent in post-perceptual thought. In this way, it has been argued that insofar as certain plausibly but non-obviously perceptual features, like number and causality, exhibit visual adaptation, they ought to be recognized as “primary visual attributes” alongside the likes of better established visual features like color, motion, and orientation (see, e.g., Burr & Ross, 2008). As Webster (2015) puts it, “Studies of these adaptations have played a long and central role in vision science, partly because the specific adaptations remain a powerful tool for dissecting vision by exposing the mechanisms that are adapting. That is, ‘if it adapts, it's there’” (Webster, 2015, p. 547). Although some have challenged this prevailing orthodoxy (see: Phillips & Firestone, 2023; Smortchkova, 2020), the idea that adaptation effects are both ubiquitous in perception and uniquely perceptual remains widely accepted, as reflected in the fact that these effects are one of the primary lines of evidence cited in support of the claim that features like number and causality are literally perceived (e.g., Burr & Ross 2008; Rolfs et al., 2013; Webster, 2015; cf., Yousif et al. 2024).

If the existence of adaptation implies that a feature is genuinely perceived, then claims about adaptation should be evaluated carefully. If nothing else, such evaluation would enable us to better appreciate the nature and scope of visual processing. In recent work, we have begun that evaluation by looking closely at *number adaptation* (Yousif et al., 2024). While we successfully replicated some classic experiments that have appeared to support the existence of number adaptation, we failed to replicate other crucial tests of its existence. Indeed, we conducted several novel experiments that put pressure on the notion of number adaptation, either because we failed to observe results that a principled theory of number adaptation should predict, or because we observed results that are strictly at odds with number adaptation. Ultimately, we concluded that claims about number adaptation should be met with skepticism – at least until more definitive evidence emerges.

In the present treatment, we take a critical look at another case study: *size adaptation*. We examine size adaptation not with the expectation that previous results will fail to obtain, but instead with the aim of understanding (a) whether evidence for size adaptation is sufficiently robust to warrant the general claim that the visual system adapts to size, and to understand (b) *how* the process of size adaptation works and in what ways it resembles other kinds of adaptation. What we find is not so much evidence that size adaptation fails to exist, but rather that it is puzzling – prone to myriad quirks and anomalies that lack an obvious explanation. To this end, we begin this paper by briefly reviewing prior work on size adaptation, before describing the results of four experiments which examine size adaptation directly, and finally discussing how the results of these experiments bear on questions (a) and (b) outlined above.

### Prior work on size adaptation

To say that the visual system exhibits *size adaptation* would be to say that, as with other visual features like color and motion, the visual system exhibits repulsive aftereffects following exposure to that feature. If you stare at a purple tree and then view a neutral stimulus, you will experience green; if you stare at a waterfall flowing downwards and then stare at a static image, you will experience upward motion. Likewise, in cases of size adaptation, if you stare at a large object and then view a smaller one, you will perceive that object to be smaller than it would otherwise appear. It is often implicitly understood that such effects are bi-directional, in the sense that staring at a green tree would also cause you to have a purple afterimage; staring at a waterfall flowing backwards would cause you to experience downward motion; and, ostensibly, staring at a small object would cause you to perceive a subsequent larger object as even larger. However, we note that not all adaptation effects are bi-directional, and it remains unclear whether it should always be expected that they are (see Yousif et al., 2024, for discussion).

**Fig. 2:**
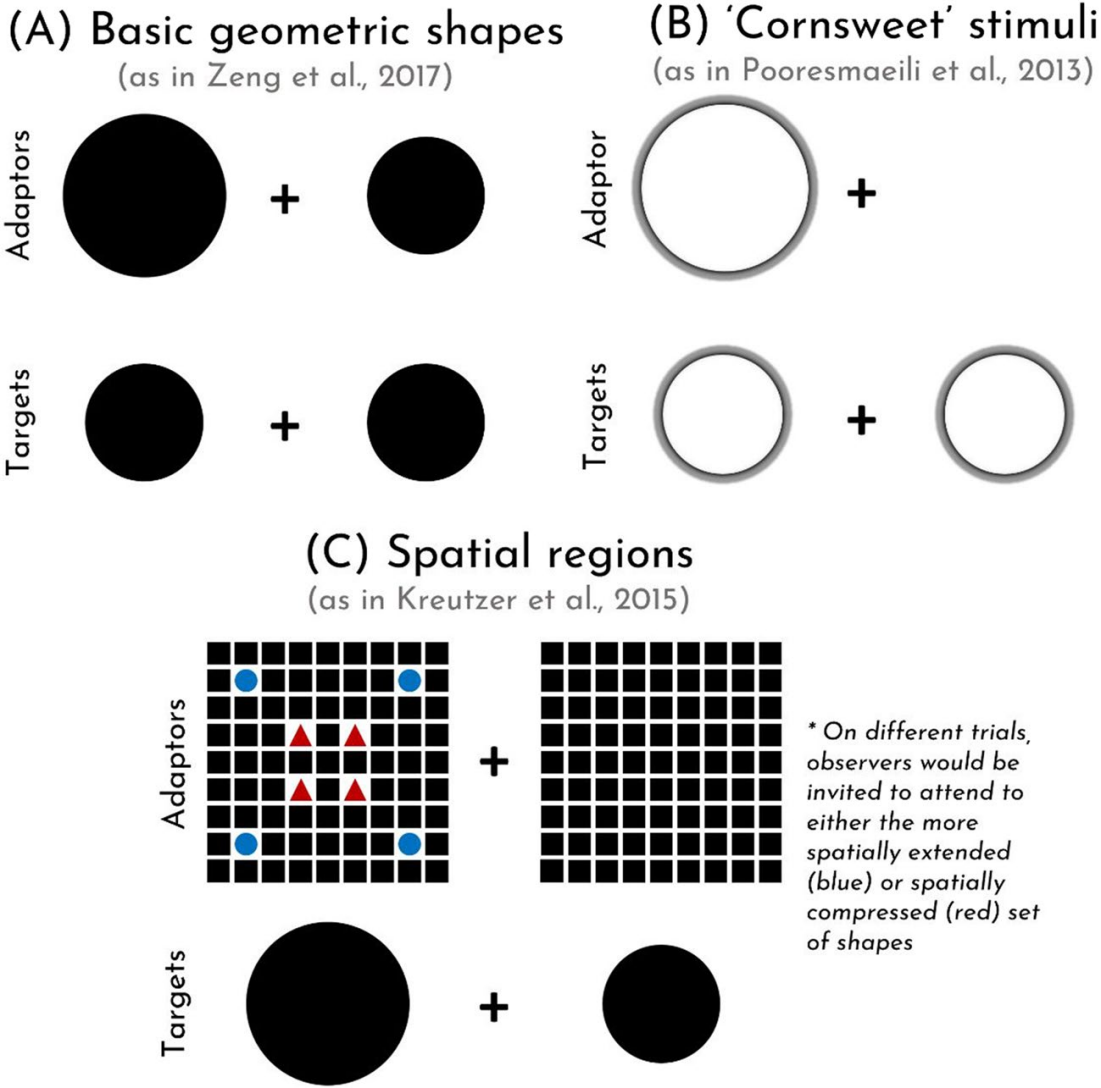
Examples of size adaptation paradigms. In this paper, we use basic geometric shapes (**A**) and ‘Cornsweet’ stimuli (**B**)

Focusing on the phenomenon of size adaptation, some have suggested that its existence might be seen as surprising, given that size is not thought to be represented in lower-level visual areas like other features that exhibit adaptation (Pooresmaeili et al., 2013). Yet, as far as we can tell, there are three ways that size adaptation effects have nevertheless been demonstrated (see Fig. 2): (1) Using simple shapes like circles, (2) using ‘Cornsweet’ stimuli with flickering borders, and (3) using areas/regions implied by multiple discrete locations (Kreutzer et al., 2015).

The first method is the most straightforward: Observers adapt to a filled in shape, like a circle, and are tested on a similar shape of a different size (see, e.g., Zeng et al., 2017; Zimmermann et al., 2016). This sort of size adaptation is the most natural in that it applies to the sorts of things that one might see as part of their ordinary experience, much as demonstrations of color and motion adaptation are striking in that they can be experienced in the real world, without the use of any carefully designed stimuli. However, an obvious drawback of these sorts of stimuli is that they may be prone to effects of contrast adaptation which problematize attempts to isolate size (e.g., Webster & Miyahara, 1997). For instance, staring at a white circle on a black background will influence the color/contrast of any subsequent object presented in the same location, regardless of its size, and it is unclear if or how this would influence perceived effects of size adaptation. Indeed, this much can be readily experienced (to see for yourself, try Demo #2).

The second method is designed to address this limitation of the first approach. By using ‘Cornsweet’ stimuli, in which uncolored objects are defined by flickering black and white edges on a neutral background, contrast adaptation is (putatively) eliminated (see Pooresmaeili et al., 2013; Tonelli et al., 2017, 2020; see Fig. 2B). The purported upshot is that any observed effect of size adaptation can be attributed to size per se rather than contrast. In prior work, researchers have found robust size adaptation effects for many such stimuli (ibid.).

The third method also purports to eliminate the effects of contrast adaptation. But it does so in a different way: by having observers adapt to regions of space defined by the arrangement of multiple items, rather than a single object (Kreutzer et al., 2015; see Fig. 2C). We’ll ignore this third method for now since it seems importantly unlike the first two, plausibly reflecting an effect of spatial attention rather than a perceptual adaptation effect of size. (This raises questions about whether the other size adaptation effects could also be understood as effects of attention in some way, but we’ll sidestep this complication for now, returning to it only in our discussion.)

### Current study

In the current study, our goal is to critically examine size adaptation. In a first experiment, we ask whether size adaptation obtains for simple geometric shapes in a setup where observers adapt to two objects at once that are either larger or smaller than target stimuli. In a second experiment, we ask whether size adaptation obtains in comparable ways when observers simply adapt to a single stimulus. In a third experiment, we specifically manipulated the color of the adaptor and target objects to assess the possible influence of color/contrast on size adaptation. In a fourth experiment, we evaluate size adaptation with ‘Cornsweet’ stimuli (which are typically expected to eliminate the effects of contrast).

To be clear, we are interested in evaluating these effects not only because they might tell us something about size perception (i.e., *whether* size is represented by the visual system, *how* it is represented in the mind, *where* it is represented in the visual hierarchy, etc.), but also because they might tell us about adaptation writ large. Questions have been raised about other “high-level” instances of adaptation, like number (e.g., Yousif et al., 2024). In fact, questions have been raised about whether adaptation is even a strictly perceptual phenomenon (as is traditionally assumed; see Helton 2016; Phillips & Firestone, 2023). If there are questions about whether adaptation is truly a marker of perceptual content (see Block, 2022; Webster, 2015), then the examination of borderline cases like number, causality, and size is likely to be informative.

With this in view, it is perhaps curious that size adaptation has received little scrutiny. Purported effects of adaptation to high-level properties, like number, have not gone unquestioned (see, e.g., Durgin 2008; Yousif et al. 2024). However, they are – by all accounts – phenomenologically striking, yielding dramatic alterations to visual appearance (see the supplementary materials of Burr & Ross, 2008). Size adaptation is, by contrast, comparably weak in both its phenomenology and behavioral effects (to illustrate, we urge readers to compare the phenomenological dramatic demos found in Burr & Ross with Demo #2–5 of the present paper). Indeed, while it strikes us that cases of adaptation to a large item *might* yield a modest (albeit questionable) effect on the perceived size of a middling target (see Demo #2), we have struggled to identify any noticeable effect of adaptation to a small item on a middling object’s size (see Demo #3), especially if other features of the stimulus are altered (see Demo #4), though we sometimes come away with the opposite impression. This lack of a clear phenomenological effect prompts us to ask: Is there sufficient evidence to license the claim that size adaptation is genuine, and, if so, are these instances of putative adaptation of the same kind as better-established, low-level forms of perceptual adaptation?

The current work is as much about phenomenology as it is about the empirical results themselves. For this reason, we have created demos that we allude to throughout this paper. Readers are encouraged to examine these demos for themselves – to consider whether their personal phenomenological impression is congruent with the results documented here.

## Experiment 1: Size adaptation with simple shapes (two adaptors)

In our first experiment, we examined a straightforward case of size adaptation. We had observers adapt to two different square objects simultaneously, one of which was always “neutral” in the sense that it was roughly the same size as the test objects. We were specifically interested in (a) whether we found a detectable size adaptation effect, (b) whether these effects were bi-directional, like color and motion aftereffects, and (c) whether they were of a comparable magnitude in each direction. Given our introspective sense that there was no detectable phenomenological alteration in the size of a middling test item following adaptation to a small adaptor and (at best) modest effects of adaptation to a large adaptor, we predicted that the observed effects would be small and, possibly, uni-directional.

### Method

For these experiments, and for all subsequent experiments in this paper, the sample sizes, primary dependent variables, and key statistical tests were chosen in advance and were pre-registered (see Open Science Framework (OSF): https://osf.io/3swph/ ).

#### Participant

Twenty participants were tested in the laboratory in exchange for course credit. One additional participant was excluded because of responses they gave during debriefing (as part of our pre-registered exclusion criteria). This study was approved by the University of Pennsylvania Institutional Review Board.

#### Stimuli

The stimuli were black squares presented on a grey background. Adaptors were presented on both sides of the screen (400 pixels offset from the center of the screen, separated by a fixation cross). One adaptor was always 100 × 100 pixels, and the other was 40 × 40, 50 × 50, 60 × 60, 180 × 180, 200 × 200, or 220 × 220 (counterbalanced across sides, resulting in 12 unique combinations). The target stimuli were also two squares (presented in the same locations as the adaptors) which varied in size. There were seven possible target stimulus combinations ([left side, right side]): [80 × 80,100 × 100], [100 × 100,120 × 120], [100 × 100,100 × 100], [100 × 100,100 × 100], [100 × 100,100 × 100], [100 × 100,80 × 80], [120 × 120,100 × 100].

#### Procedure

Participants were introduced to the task via brief verbal instructions. They were shown an example of a typical size adaptation trial, in which it was explained that they would be judging which of two squares was larger. Afterward, they were allowed to ask questions about the procedure before beginning the task. On each trial, the adaptors appeared for 10 s, after which the target stimuli appeared for 750 ms. Unlike prior studies of this kind, we elected not to include a blank screen in-between the adaptor and target stimuli. We did this for three reasons: (1) Canonical instances of adaptation like color adaptation and motion adaptation do not seem to depend on such blank screens; (2) We are not aware of any reason to think that blank screens do or ought to influence adaptation effects; and (3) We struggle to identify principled reasons to include blank intervals of any specific duration. Participants were tasked with indicating which of the two target stimuli was larger. They pressed “Q” if the left square was larger or “P” if the right square was larger. As there were 12 adaptor size combinations and seven target size combinations, there were 84 unique trials. The trials were presented in a random order for each participant.

### Results and discussion

**Fig. 3:**
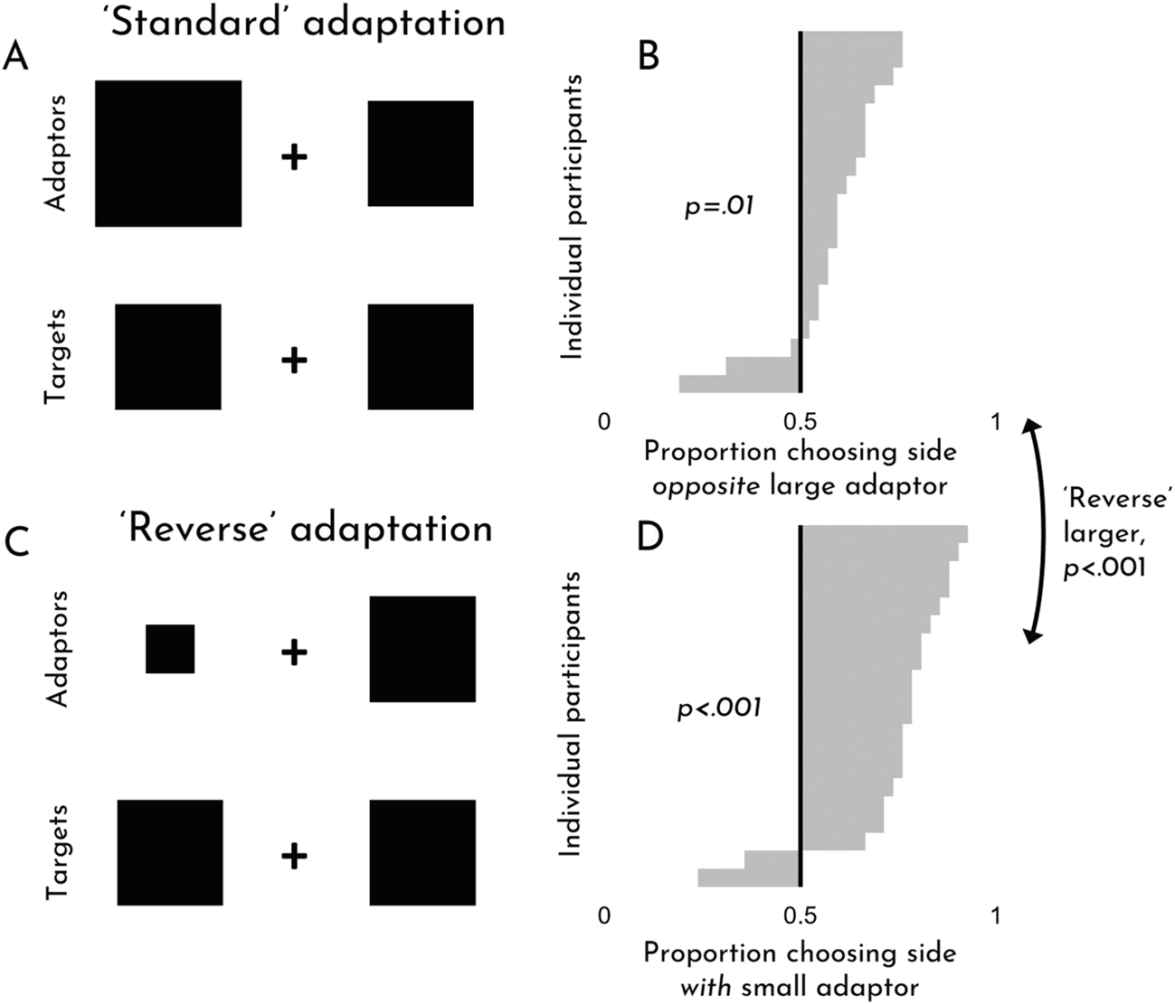
Experiment 1. An example of standard adaptation trial (**A**) and a ‘reverse’ adaptation trial (**C**). The effects for each trial type, broken down by participant (**B, D**). Examples are for demonstration purposes; items are not to scale

The results of Experiment 1 can be seen in Fig. 3. As is evident from the figure, we observed robust size adaptation effects for both standard adaptation (*M* = .59, *SD* = .14; *t*(19) = 2.78, *p* = .012, *d* = .62; the mean here refers to the proportion of trials for which participants selected the side opposite the larger adaptor) and ‘reverse’ adaptation, wherein observers adapted to a small item before being tested on a mid-sized item (*M* = .75, *SD* = .17; *t*(19) = 6.57, *p* < .001, *d* = 1.47; the mean here refers to the proportion of trials for which participants selected the side with the smaller adaptor). Counter to our pre-registered expectation, however, we found that ‘reverse’ adaptation effects were *stronger* than standard adaptation effects (*t*(19) = 7.01, *p* < .001, *d* = 1.57). For good measure, we also checked whether people were sensitive to the different sizes of the target stimuli. They were. Perhaps unsurprisingly, participants selected the item that was actually larger the vast majority of the time (*M* = .81, *SD* = .10; *t*(19) = 13.87, *p* < .001, *d* = 3.10).

This larger effect for ‘reverse’ adaptation compared to standard adaptation is striking, since it runs counter to our introspective assessment of size adaptation’s magnitude and phenomenology, given that we struggled to appreciate any phenomenologically noticeable effect of reverse adaptation (compare again Demo #2 and Demo #3). For many phenomena, such phenomenological effects could be considered a bonus, rather than a requirement. But in standard cases of visual adaptation, it’s natural to think of the phenomenology *as* the phenomenon. That the apparent phenomenology of these effects was a poor guide to the strength of the observed effects, suggests that this commonsense assumption should be handled with caution: *we do not simply know cases of size adaptation when we see them*. Rather, the connection between phenomenology and behavior is tenuous, or so it would seem.

An anonymous reviewer asked us about the limitations of a dichotomous response scheme (i.e., in which participants are forced to indicate that one object is larger than the other). Incidentally, we had already run a similar version of this experiment, in which participants also had an option to indicate that both sides were equal. Though participants used the neutral option choice about 18% of the time overall (and 23% of the time on trials in which the targets were truly equal), we nevertheless observed significant adaptation in both cases. However, unlike in the original experiment, standard adaptation exhibited a larger effect (*t*(19) = 3.47, *p* = .003, *d* = .78). The full data are included in the data file on our OSF page as Experiment S1 (OSM) and summary figures can be seen in Fig. S1 (OSM).

## Experiment 2: Size adaptation with simple shapes (one adaptor)

Our second experiment was identical to Experiment 1, but with a twist: Participants only adapted to a single object. Neutral adaptors were removed. By removing the neutral adaptor, we can evaluate whether adapting to multiple objects matters. There are good reasons to think that it might. Prior work has shown that number adaptation, but not orientation adaptation, is influenced by “implicit visuospatial attention” (which, in this case means the presence of a second adaptor; see Grasso et al., 2021). Prior work has also shown that ‘reverse’ number adaptation depends in a critical way on the presence of a neutral adaptor (Yousif et al., 2024). Examining whether size adaptation is similarly influenced by the presence of a second adaptor may, therefore, help us to better understand the mechanisms underlying size adaptation.

### Method

This experiment was identical to Experiment 1 except that all neutral adaptors were removed. Participants adapted to a single object but still compared the relative size of two target objects. Twenty unique participants completed this task, and an additional one was excluded because of responses given during debriefing.

### Results and discussion

**Fig. 4:**
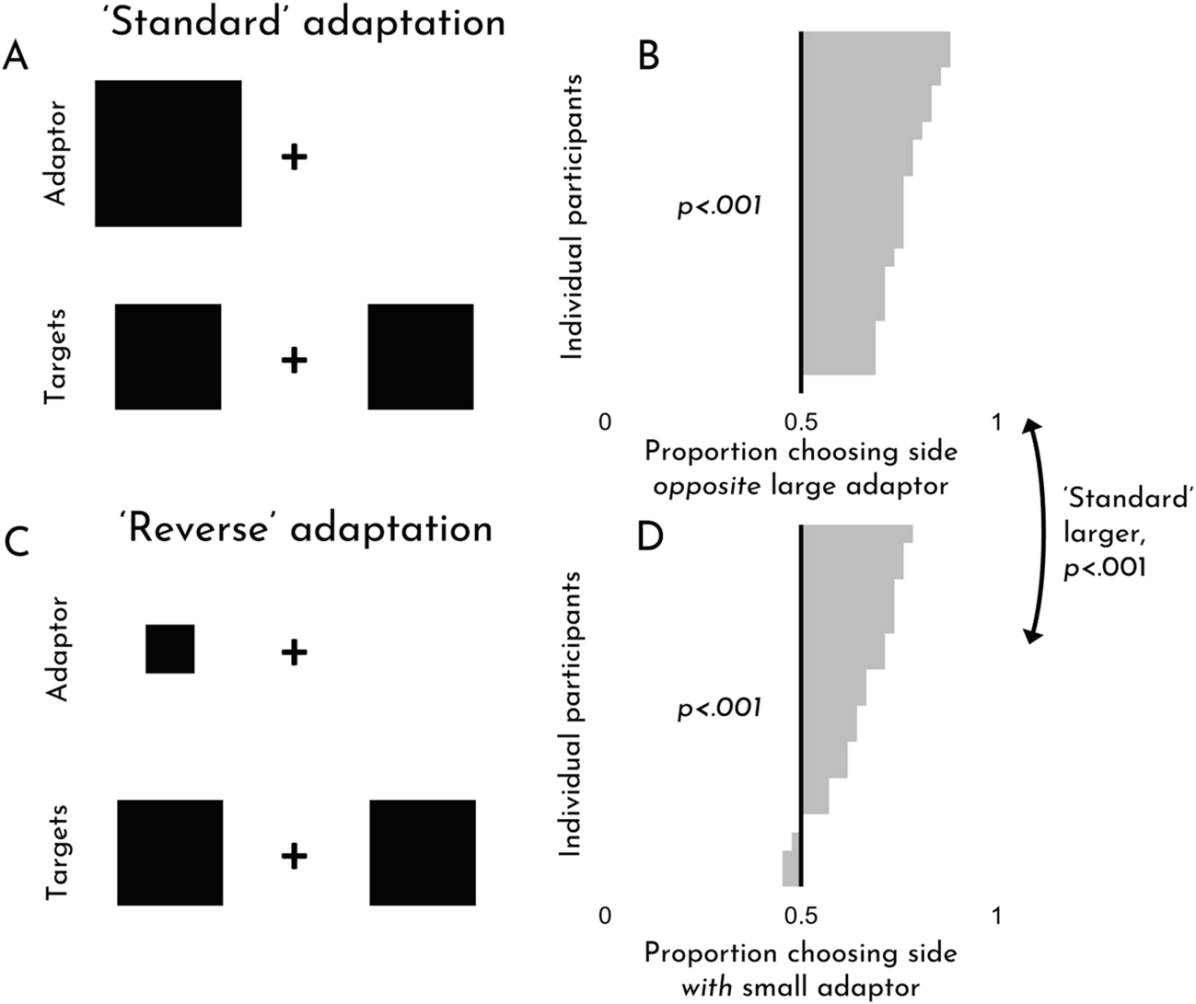
Experiment 2. An example of a standard adaptation trial (**A**) and a ‘reverse’ adaptation trial (**C**). The effects for each trial type, broken down by participant (**B, D**). Examples are for demonstration purposes; items are not to scale

The results of Experiment 2 can be seen in Fig. 4. As is evident from the figure, we observed robust size adaptation effects for both standard adaptation (*M* = .78, *SD* = .06; *t*(19) = 19.19, *p* < .001, *d* = 4.29) and ‘reverse’ adaptation (*M* = .65, *SD* = .10; *t*(19) = 6.60, *p* < .001, *d* = 1.48). Consistent with our pre-registered expectation, but in contrast with Experiment 1, we found that ‘reverse’ adaptation effects were *weaker* than standard adaptation effects (*t*(19) = 4.51, *p* < .001, *d* = 1.01). Once again, people correctly chose the target objects that were larger when appropriate (*M* = .81, *SD* = .08; *t*(19) = 18.13, *p* < .001, *d* = 4.05).

The asymmetry between standard and reverse adaptation is non-trivial. In Experiment 1, reverse adaptation effects were substantially larger; and here, standard adaptation effects are substantially larger. For that reason, we feel that this asymmetry is worth highlighting and exploring further. As things stand, we know of no published explanation for this asymmetry. Nevertheless, it seems clear that an adequate account of size adaptation should offer one. This lays down a challenge for future theoretical work in the area.

## Experiment 3: Size adaptation with simple shapes (color swaps)

A potential problem with testing size adaptation using simple shapes is that such figures create noticeable afterimages (i.e., color/contrast adaptation). This is not surprising, given that color adaptation effects are ubiquitous and powerful (as readers were invited to experience for themselves at the start of this paper). It is not clear, however, to what extent the previously observed adaptation effects hinge on contrast adaptation. To get a handle on this, we borrowed a design from a prior study of ours in which we ran a standard number adaptation task but swapped the colors of co-localized items between the adaptation phase and the test phase (Yousif et al., 2024). We found that swapping the colors of items significantly reduced the adaptation effect. Similar work demonstrated that swapping the colors in a number adaptation task *fully* eliminated the adaptation effect (Grasso et al., 2022). So, here we asked: Will a color swap similarly reduce or eliminate size adaptation effects?

### Method

This experiment was identical to Experiment 2 except as stated below. Twenty unique participants completed this task; there were no exclusions.

There were only two differences between this experiment and Experiment 2. First, we reduced the range of possible adaptor sizes. On each trial, the adaptor was either half the average size of the target stimuli (50 × 50) or double the average size of the target stimuli (200 × 200). The possible sizes of the target stimuli remained the same.

Second, and more critically, the color of the targets/adaptors was not constant throughout the task. On half of the trials, the adaptors and targets were the same color (half of the time black; half of the time white). On the other half of the trials, the adaptors and targets were opposite colors (half of the time the adaptors were black and the targets white; half of the time the adaptors were white and the targets black).

As there were four adaptor size combinations (small-left, small-right, large-left, large-right), seven target size combinations, and four color combinations (white/white, black/black, white/black, black/white) there were 112 unique trials.

### Results and discussion

**Fig. 5:**
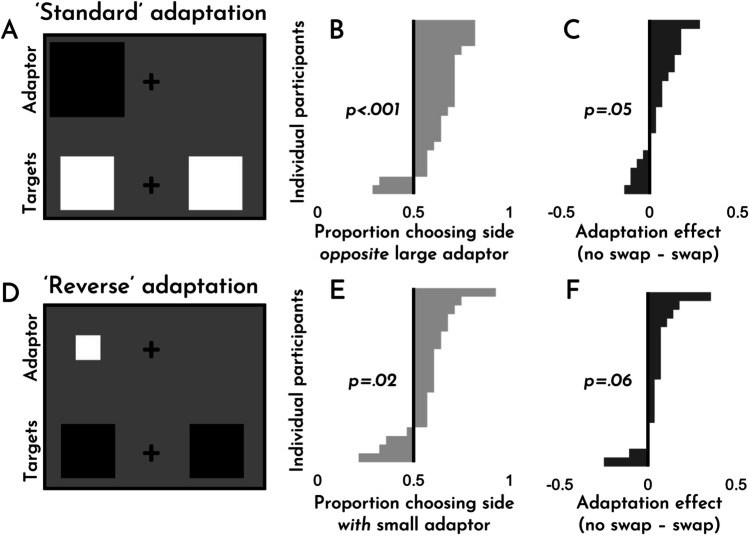
Experiment 3. An example of a standard adaptation trial (**A**) and a ‘reverse’ adaptation trial (**D**). The effects for each trial type, broken down by participant (**B, E**). The difference in the magnitude of the adaptation effect depending on whether the colors swapped or did not swap, broken down by participant (**C, F**). Bars to the right of the axis indicate a stronger effect when the colors did not swap. Examples are for demonstration purposes; items are not to scale. Detailed results for each trial type can be seen in Fig. S3 in the Online Supplemental Material on the Open Science Framework page

The results of Experiment 3 can be seen in Fig. 5 (and more detailed results can be seen in Fig. S2 (OSM)). As is evident from the figure, we observed robust size adaptation effects for both standard adaptation (*M* = .65, *SD* = .14; *t*(19) = 4.77, *p* < .001, *d* = 1.07) and ‘reverse’ adaptation (*M* = .59, *SD* = .16; *t*(19) = 2.46, *p* = .024, *d* = .54), even when the colors swapped between the adaptation phase and test phase. However, we also found that adaptation effects were significantly weaker overall when the colors swapped as opposed to when they did not (*t*(19) = 4.04, *p* < .001, *d* = .90). This difference suggests that color/contrast adaptation likely plays some role in size adaptation with simple geometric shapes (and in this way resembles the fact that number adaptation effects are reduced or eliminated when the color of stimuli changes between adaptation and test; see Grasso et al., 2022; Yousif et al., 2024).

## Experiment 4: Size adaptation with ‘Cornsweet’ stimuli (one adaptor)

Experiment 3 indicated that afterimages likely play some role in size adaptation effects for simple geometric shapes. However, as we discussed at the beginning of this paper, there are multiple kinds of stimuli that have been used to evaluate size adaptation. One proposed solution to the problem of contrast adaptation is to use what we call ‘Cornsweet’ stimuli — essentially discs with alternating, flashing black and white edges (see Pooresmaeili et al., 2013; Tonelli et al., 2017, 2020). Though never articulated directly, these stimuli putatively eliminate concerns about contrast adaptation by ensuring that the visual system is never adapting to any one color in any one location; the objects themselves have no color, and the edges that define them constantly alternate colors. As such, ‘Cornsweet’ stimuli provide a powerful means of probing whether putative size adaptation pertains to size *itself*. Here, we asked whether we would observe robust size adaptation effects using these stimuli in a setup identical to the ones used in our previous experiments.

### Method

**Fig. 6:**
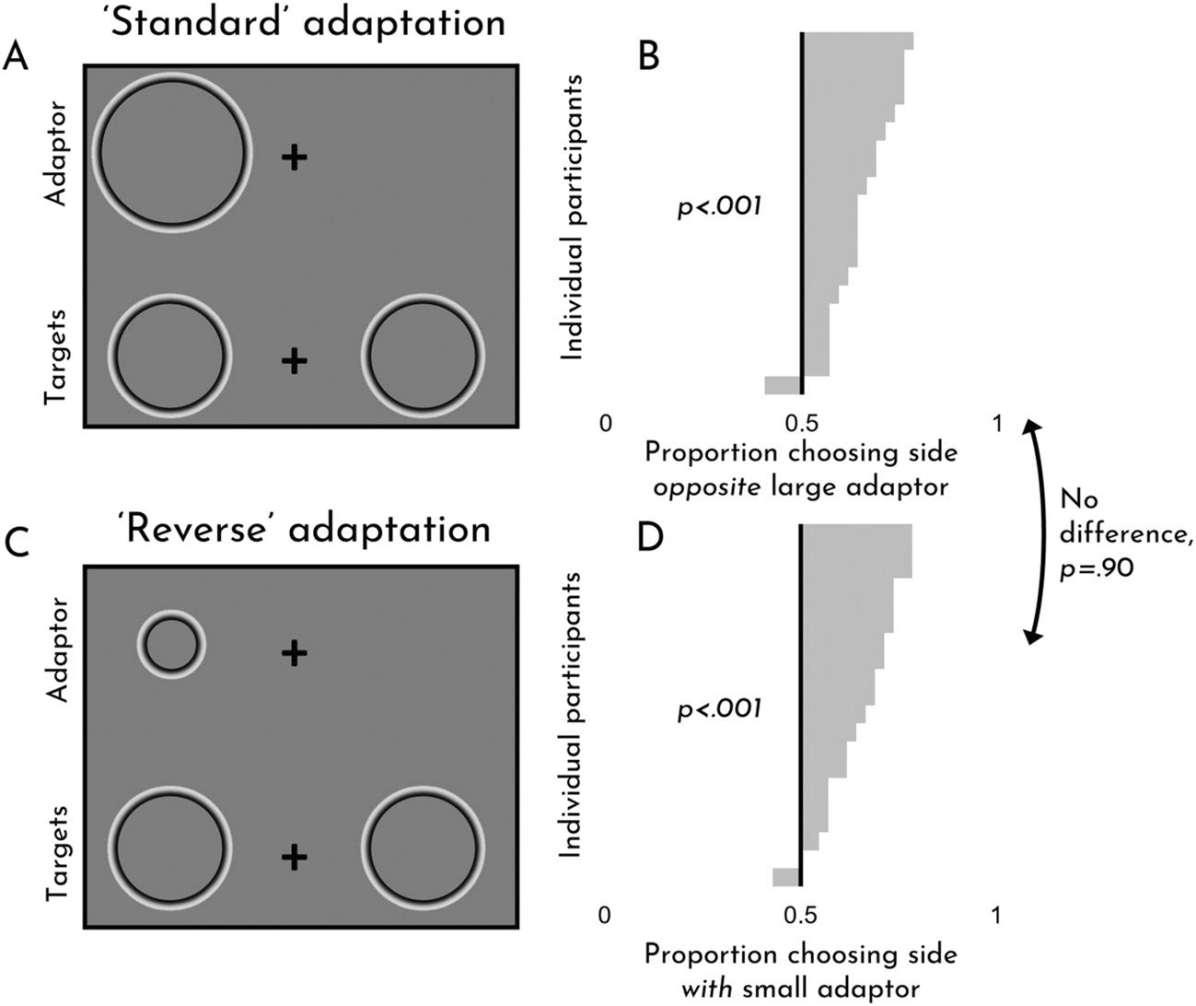
Experiment 4. An example of standard adaptation trial (**A**) and a ‘reverse’ adaptation trial (**C**). The effects for each trial type, broken down by participant (**B, D**). Examples are for demonstration purposes; items are not to scale

This experiment was identical to Experiment 2 except that we used ‘Cornsweet’ stimuli rather than simple geometric shapes (see Fig. 2B and 6; see Demo #5). In our implementation, the stimuli flickered between black and white lines every frame (60 Hz) on a middling grey background. To accommodate these different stimuli (because the flickering stimuli were difficult to appreciate at the smallest sizes), we doubled the size of all stimuli across the board (such that, e.g., an adaptor that would have been 200 × 200 pixels would become 400 × 400 pixels). Twenty unique participants completed this task, and an additional one was excluded because of erratic response times during the task.

### Results and discussion

The results of Experiment 4 can be seen in Fig. 6. As is evident from the figure, we observed robust size adaptation effects for both standard adaptation (*M* = .65, *SD* = .09; *t*(19) = 7.42, *p* < .001, *d* = 1.66) and ‘reverse’ adaptation (*M* = .66, *SD* = .10; *t*(19) = 6.94, *p* < .001, *d* = 1.55). Unlike Experiments 1 and 2, we did not observe any asymmetry between standard and reverse adaptation (*t*(19) = .12, *p =* .90, *d* = .03). These results demonstrate that bi-directional size adaptation with ‘Cornsweet’ stimuli is robust, putting pressure on the notion that size adaptation could be fully explained by color/contrast adaptation. Once again, this is surprising since the phenomenology of these effects is subtle and, at times, entirely non-obvious. We discuss this matter in greater detail in the discussion that follows.

As with Experiment 1, we ran a version of this experiment in which participants had the option to indicate that both sides were equal. Participants selected this option about 26% of the time overall, and as much as 42% of the time on trials in which the targets were equal in size. These values suggest that even though participants are exhibiting a size adaptation effect, it is modest enough that they report seeing nothing on almost half of the relevant trials. The full data are included in the data file on our OSF page as Experiment S2 (OSM) and summary figures can be seen in Fig. S3 (OSM).

## General discussion

We have shown that size adaptation effects are robust across a range of experimental approaches, whether observers adapt to two objects (Experiment 1) or one (Experiment 2), whether the color of the adaptors and targets are matched or not (Experiment 3), and, perhaps most compelling of all, with ‘Cornsweet’ stimuli which are designed to reduce or even eliminate effects of contrast adaptation (Experiment 4).

We have, however, also shown that there are unanswered puzzles of size adaptation. For instance, we found that there are larger effects of reverse size adaptation than canonical size adaptation when observers adapt to a neutral stimulus on one side of the display. Yet we found the exact opposite when observers adapt to only a single stimulus (and no neutral stimulus). Additionally, we found that while there are still effects of size adaptation when the adaptors/targets are different colors, these effects are weaker than when they are of the same color. Finally, we noted that despite yielding phenomenologically underwhelming illusions of size when using ‘Cornsweet’ stimuli (see Demo #6), robust size adaptation effects persisted under these conditions.

These findings raise a host of unanswered questions. That the magnitude of size adaptation effects depends on the presence of a neutral adaptor implies that the neutral adaptor is causally relevant. This is not what is typically assumed. Typically, it is assumed that two objects of equal size should not influence each other (here, there should be no opportunity for a repulsive effect from a neutral adaptor on its target, given that the values are the same). Moreover, adaptation is typically viewed as a spatially isolated repulsive aftereffect, meaning that a stimulus of a given value should cause any differing subsequent stimulus to be perceived as farther from that value only when presented in the corresponding region (a fact that is actually not true for size adaptation; see Altan & Boyaci, 2020). What our results thereby suggest is that some factor other than size (at a given location) is influencing the observed adaptation effect. If that is true, then worries arise as to how ‘size’ could ever be isolated. At best, the strength of size adaptation to a given stimulus becomes hard to quantify; at worst, it may even become hard to establish that any given effect pertains to size adaptation rather than orthogonal properties of the display.

One factor that likely influences the impression of size adaptation is contrast. Under normal circumstances, we know that observers adapt to color (Webster, 1996). Staring at a black square should influence the perception of a subsequent object. Though it remains unclear how exactly this would influence size adaptation, it is clear that color/contrast adaptation likely plays some role. This was evidenced by the fact that changing the color of the adaptors/targets reduces the magnitude of the size adaptation effect (Experiment 3). Furthermore, the effects of contrast can be readily appreciated for oneself when using standard size adaptation stimuli (see Demo #2).

Another factor that might influence size adaptation is adaptation to the presence of objects themselves: Recent work has argued, for instance, that the visual system may adapt to the presence of objects in such a way that unchanging items will sometimes be filtered out from awareness (Yousif et al., 2024). If true, this could help explain why a neutral adaptor had an effect in Experiment 1; it may be causing the subsequent neutral target to be partially filtered from awareness. Such filtering might even explain the asymmetry that we observed (i.e., that reverse adaptation is more pronounced when there is a neutral adaptor, but standard adaptation is more pronounced when there is not), though this remains largely speculative for now.

Size adaptation using ‘Cornsweet’ stimuli (Experiment 4; see also Pooresmaeili et al., 2013; Tonelli et al., 2017, 2020) is meant to avoid these problems. As such, it is telling that we observe both standard and reverse size adaptation effects when using such stimuli; this fact alone seems to be a compelling reason to believe that size adaptation is genuine. However, it isn’t clear that the ‘Cornsweet’ method is immune to critique.

There are at least three and a half reasons why we think experiments with ‘Cornsweet’ stimuli should be interpreted with caution. First: These effects are not as phenomenologically compelling as other canonical adaptation effects, like motion adaptation or color adaptation (which readers were invited to experience for themselves when examining Fig. 1). The phenomenological experience of size adaptation with ‘Cornsweet’ stimuli is modest at best. For most phenomena in cognitive science, the presence of a robust empirical difference is meaningful on its own – but adaptation is not like most phenomena. Adaptation might be unique in the sense that its significance is often tied to its phenomenology. The reason that students “Ooh” and “Aah” when they see such demonstrations for the first time is because they really do *see* them. They perceive them in a manner that is so compelling that they may sense they are being tricked. Seeing them a second time, and a third time, and a fourth time, they are finally forced to admit that the illusions are indeed real. In these demonstrations of size adaptation, one might have the vague sense that the size of any object is perceptibly different, but these demos hardly evoke a sense of astonishment.

In fact, when participants are given an opportunity to offer a neutral response (i.e., indicating that neither side looks larger than the other, in Experiments S1 and S2 (OSM)), they did so as much as 42% (!) of the time (on trials in which the targets are truly equal in size). This means in practice that the illusion is so underwhelming that participants reported experiencing no difference nearly half the time. This fact alone raises questions about how robust this phenomenon is in the first place.

The second reason size adaptation effects should be interpreted with caution is because it isn’t entirely clear that ‘Cornsweet’ stimuli solve the problem they are meant to solve. To the best of our knowledge, it has never been stated exactly how ‘Cornsweet’ stimuli escape concerns of image aftereffects and low-level confounds of contrast adaptation; the reader is simply asked to take that fact for granted. Yet insofar as the flashing edges of the stimulus defines an object, that object may appear to have a color slightly different from the background. Indeed, one easily recognizes the ‘Cornsweet’ object as one that is distinct from the background (rather than seeing this as a collection of flickering edges that are not filled in). Whether this could cause some modest afterimage, we are not sure. We are sure, however, that this assumption should be carefully evaluated.

A third reason that size adaptation effects should be interpreted with caution is that it remains unclear whether they involve adaptation to size per se. In the beginning of this paper, we mentioned three different ways that size adaptation effects have been demonstrated (see Fig. 2). One of those involves asking observers to attend to a larger or smaller region of space, but *not* to the size of an object itself (see Fig. 2C; Kreutzer et al., 2015). We would argue that these effects are naturally understood as effects of spatial attention (i.e., that there is some kind of repulsive aftereffect such that attending broadly subsequently causes attention to narrow, and vice versa). One might think that a repulsive aftereffect on spatial attention is tantamount to a repulsive aftereffect on size insofar as they yield the same result. But the difference, we think, is important, since it concerns a distinct medium over which the adaptation occurs. For if size aftereffects arise from attention alone, it is unclear whether it would still be fair to say that the visual system is adapting to the represented dimension of size. Certainly, such a result would have no obvious consequences for traditional questions concerning the represented contents of perception, given that attentional states are not naturally thought of as representational states (Koralus, 2014; Mole, 2011) yet remain subject to top-down cognitive influences. (The same is, of course, true for other adaptation effects; adaptation to number is not meaningful in this context unless adaptation is happening on the represented dimension of number; otherwise, claims about the meaningfulness of adaptation, including the fact that adaptation to a feature implies that it is perceptually represented, are unwarranted; see Yousif et al., 2024.)

But if it is true that some effects of size adaptation are naturally thought of as aftereffects on spatial attention, it is not immediately clear what prevents that same interpretation applying to other stimuli, including ‘Cornsweet’ stimuli. That these might be susceptible to a similar analysis is even suggested by work showing that eye movements to the edge of a stimulus are influenced by adaptation (as if participants are literally looking for its edges in a different location; Zimmermann et al., 2016). This is an intriguing finding in its own right: It could mean that there are repulsive size aftereffects, but that these arise as an effect of attention rather than as a consequence of participants’ adaptation to visual representations of size per se. But, as explained above, this problematizes the interpretation of such effects, at least insofar as they are meant to inform debates concerning the contents of human vision.

Some work has specifically argued that size adaptation is *not* influenced by attention (Tonelli et al., 2020), which might seem to undermine the suggestion that spatial attention plays a crucial role in these phenomena. However, we’re not sure that the same kinds of attention are at play here. Tonelli and colleagues manipulated attention via taxing distractor tasks; here, we are concerned with the spread of attention across space. That said, we acknowledge that there is some ambiguity about the role that attention plays in size adaptation.

A final, albeit speculative, (half) reason to handle size adaptation to ‘Cornsweet’ stimuli with caution concerns the fact that size perception is poorly understood. Not only are there numerous illusions of size (see Coren & Girgus, 1978), size perception is said to be illusory even under normal viewing conditions (see Bennette et al., 2021; Yousif & Keil, 2019, 2021). Illusions of size are so ubiquitous that simply rotating an object can dramatically alter its perceived size: A square rotated 45° (i.e., a diamond) is perceived as larger than a square in its canonical orientation, for instance (Yousif et al., 2020). Size is also notoriously underdetermined by visual input: A skyscraper will project only a small image on the retina if it is far away, while a fly will project a large visual image when viewed close up. But given that the visual system utilizes both retinal size and physical size in the course of its computations (see Long & Konkle, 2017), it isn’t obvious over which units size adaptation would or should operate. Furthermore, if a diamond is perceived as larger than an equivalent square, does that mean that we should expect those two shapes to induce adaptation in each other? We aren’t sure. We would argue, however, that this lack of clarity complicates the interpretation of documented size adaptation effects.

### The fragility of size adaptation

Despite convincing evidence in support of size adaptation, such effects appear to be fragile in ways that have not been acknowledged previously. For instance, we have shown that there is a surprising asymmetry between cases of standard and reverse size adaptation depending on whether observers simultaneously adapt to a neutral adaptor or not. Such quirks drive home the point that we currently possess a poor understanding of the phenomenon. In the previous section, we proposed two factors that may influence the magnitude of size adaptation effects: color/contrast and the presence of objects themselves. But there are other possibilities. In the context of number adaptation, for instance, it has been shown that the magnitude of number adaptation effects varies depending on whether observers adapt to a single adaptor or to two adaptors (Grasso et al., 2021). This difference has been explained by appeal to a difference in implicit visuospatial attention, which putatively influences number adaptation but *not* orientation adaptation. Perhaps the same could be said here: It may well be that the presence of a second, neutral adaptor is influencing attention in some way that causes the asymmetry we observe. It seems to us that all of these possibilities – influences of color/contrast, adaptation to the presence of objects, and attention – remain tenable.

We should care about such possibilities because we should care about what adaptation is. As of writing this paper, there are documented effects of adaptation to at least a dozen different visual features, including orientation, motion, speed, number, causality, and even facial features (see Fig. 1B). Are all of these adaptation effects of the same fundamental kind? Do they obtain under all the same circumstances? Do they work in the same way? If not, what are the differences – and at what point is something no longer ‘adaptation’ in the relevant way?

If adaptation effects are *not* of the same fundamental kind (perhaps because some cases of ‘adaptation’ are influenced by attention in ways that others are not; Grasso et al., 2021; or because some are retinotopic while others are not), then we should be cautious when generalizing from one case to another, as when we conclude that adaptation to a high-level feature is perceptual because adaptation to color or orientation is (see also Smortchkova, 2020). After all, number adaptation, causality adaptation, and size adaptation may not depend on visual processing in the ways that motion adaptation and color adaptation do. And other adaptation-like effects (like random number generation – Phillips & Firestone 2023; or “prevalence-induced concept change”; see Levari et al., 2018) should not be dismissed as fundamentally distinct merely because they are not perceptual in nature.

If any positive evidence is taken as definitive evidence of adaptation and inconsistent results are ignored, then we risk an endless proliferation of adaptation and adaptation-like effects. This should be resisted. Given the importance of adaptation to our understanding of the mind and brain’s basic organization, the standards for labeling something ‘adaptation’ should be high. That is why, despite good reasons to believe that size adaptation is genuine, we think it useful to look at the phenomenon through a skeptical lens.

### Looking ahead

What should be done to better understand size adaptation? First, we think it is worth clarifying which “size adaptation effects” reflect genuine size adaptation. We’ve shown here that the most basic possible size adaptation paradigm, in which observers adapt to one colored object and are tested on another, should probably be avoided. It is difficult in such a setup to rule out the influence of contrast adaptation. The data in Experiments 1–3 validate this concern, insofar as these effects seem highly contingent on the color of the stimuli as well as the presence of a neutral adaptor. We also briefly discussed a size adaptation paradigm in which people adapt not to individual objects, but to regions that are bounded by individual objects. For reasons we foreshadowed before, we think this approach should also be avoided. ‘Cornsweet’ stimuli, used in some prior work (see Pooresmaeili et al., 2013; Tonelli et al., 2017, 2020) and in Experiment 4, seem like the most promising way forward. They offer to address concerns about contrast adaptation and seem to yield size adaptation effects regardless. In addition, it is worth noting that this was the one case where reverse size adaptation and canonical size adaptation effects were of similar magnitude. Still, as we articulated in the previous section, there are reasons to be unsure of whether these demonstrations of size adaptation are bulletproof.

Second, and related to the previous points, we should attempt to clarify the role that phenomenology plays in motivating or establishing the existence of adaptation. What should be made of the fact that, in Experiment 4, we observe empirically robust adaptation effects while the demos themselves seem so underwhelming?

If phenomenology is *not* a critical part of adaptation, then perhaps we need to expand our notion of what adaptation is. There are many other phenomena that could be characterized as ‘repulsive aftereffects’ that are currently not labeled as adaptation effects, in part because they do not yield readily appreciable effects on perceptual phenomenology. Take something as distant from perception as random number generation (see, e.g., Phillips & Firestone, 2023). It is well known that, when generating sequences of random numbers, people will not only resist repeating the same number successively, they will also resist selecting from the same side of the distribution (albeit to a smaller extent; see Yousif et al., 2022). What, but phenomenology, prevents us from calling this an adaptation effect?

This brings us to our third point: Size adaptation effects are not like other known instances of adaptation. For instance, though it is rarely acknowledged, size adaptation is *not* retinotopic (Altan & Boyaci, 2020); perceiving an object at one point in space influences the evaluation of an object’s size at other regions of space. Other putative cases of high-level adaptation, like adaptation to number (Arrighi et al., 2014) and causality (Kominsky & Scholl, 2020), are also not strictly retinotopic. This is odd insofar as retinotopic adaptation is typically taken as a defining property of genuine adaptation – or, at least, a necessary condition for these effects licensing claims about the contents of perception (but see Melcher, 2005). This view has been articulated in no uncertain terms. Kominsky and Scholl (2020) write, for instance, “one indication that many such forms of adaptation must reflect visual processing per se is simply that many of these types of adaptation… operate retinotopically” (p. 3). They go on to say that this is not only true, but “largely unambiguous and uncontroversial” (p. 3). What should be made, then, of the fact that many newly discovered kinds of high-level adaptation are unambiguously non-retinotopic?

There are other ways that adaptation effects like these are heterogeneous. It is unclear, for example, whether adaptation effects are, or ought to be, influenced by attention. Anton-Erxleben and colleagues (Anton-Erxleben et al., 2013) argue that attention and speed have independent effects on adaptation, such that attention always increases perceived speed, whereas adaptation only sometimes decreases perceived speed. A meta-review by Bartlett and colleagues (Bartlett et al., 2019) argues that motion adaptation, too, is influenced by attention to a large degree. Likewise for number: Grasso and colleagues (Grasso et al., 2021) argue that number adaptation *is* shaped by “implicit visuospatial attention,” but that other kinds of adaptation, namely orientation adaptation, are not. For some features, it is simply unclear whether attention plays a critical role. For instance, there are conflicting findings regarding how attention influences motion adaptation (Morgan, 2013; Morgan & Solomon, 2019; Rezec et al., 2004). As for size adaptation, Kreutzer and colleagues (Kreutzer et al., 2015) argue that size adaptation is influenced by attention, whereas Tonelli and colleagues (Tonelli et al., 2020) have argued size adaptation is not influenced by attention at all: Size adaptation effects are just as large when observers experience adaptation while completing a demanding attention task as they are when only focusing on the adaptation itself. This is to say nothing of the fact that for some kinds of adaptation, like color adaptation, it isn’t even clear what it would mean for attention to influence adaptation. (When one views a canonical color adaptation demo, the effects seem to occupy the entire visual field, but also do not seem to critically depend on any sort of explicit attention.) What’s troubling here isn’t merely the heterogeneity of adaptation effects, but the fact that there seems to be no consensus about whether attention *should* influence adaptation. In fact, we’re not aware of any arguments one way or the other. The clearest grappling with this issue that we’re aware of comes from Anton-Erxleben and colleagues (Anton-Erxleben et al., 2013), who argue that effects of attention on speed adaptation undermine the traditional assumption that adaptation is merely a by-product of neuronal fatigue. This is a rather deep issue, which should be explored further, since it bears upon vexed theoretical issues. For instance, the reasons we would have for deeming adaptation effects distinctively perceptual and differentiable from (e.g.) random number generation or prevalence induced concept change.

Finally, we should confront head-on the possibility that task demands play a role in adaptation experiments like these. In the absence of visually appreciable phenomenology to these adaptation effects, we cannot rule out the possibility of response bias. Experiments like ours do not exactly disguise their purpose; participants can easily guess that relative size plays a role in them. (We asked participants after each experiment what they thought the experiment was about, in an open-ended way; the majority of them commented on the relative size of the adaptors/targets.) Tasks demands can be insidious at times, and we should be conservative when interpreting effects that could plausibly be explained by them (see Firestone & Scholl, 2016; see also Yousif et al., 2024). This is especially true insofar as size adaptation is shown to be non-retinotopic and perhaps even non-spatiotopic (Altan & Boyaci, 2020); this fact alone seemingly opens the floodgates for cognitive accounts of size adaptation effects.

In other words, there is work to be done pinning down not only whether size adaptation is genuine and robust, but also how it works. It is important to understand why neutral adaptors influence responses. It is important to understand why a change in color influences the magnitude of size adaptation (and why the same is true for number adaptation; Grasso et al., 2022; Yousif et al., 2024). And it is important to interrogate whether or not the varied methods that have been employed to study size adaptation in prior work (see Fig. 2) are all capturing phenomena of the same fundamental kind.

This paper does not represent an exhaustive treatment of size adaptation. There are many opportunities to investigate the phenomenon further. One clear limitation of the current studies, for instance, is that we have not attempted to quantify the *magnitude* of size adaptation; we have instead focused on broader, qualitative comparisons (e.g., highlighting the difference between standard and reverse size adaptation effects in Experiments 1 and 2). While we think the broad-brush approach that we’ve taken here is valuable, there’s plenty of room for nitty-gritty psychophysical approaches to further enhance our understanding of these effects.

## Conclusion

Adaptation is often heralded as a key factor delineating “the border between seeing and thinking” (Block, 2022; see also Webster, 2015). In this way, size adaptation, and the class of related adaptation effects, promises to provide a window onto the basic mechanisms and representations of visual perception. Whether adaptation should be held in such high regard, however, may depend on what we make of the growing body of adaptation effects. Are they all of the same kind? Do they all share the same compelling phenomenology? Should we care if they do not? While the present study has replicated several basic size adaptation effects, we have also shown some such effects that are not easily explained by the conventional understanding of adaptation as a phenomenologically striking, and straightforwardly repulsive aftereffect. We have further argued that these less easily explained effects are not just curiosities, but substantive challenges to our understanding of size adaptation. Future work – examining size adaptation as well as other instances of high-level adaptation – should seek to explain the full range of these effects.

## Data Availability

Pre-registrations and raw data for all experiments are available on our OSF page: https://osf.io/3swph/

## Code availability

Code for experiments as well as analyses will be immediately made available upon request, with no exceptions.
